# De Novo *PORCN* and *ZIC2* Mutations in a Highly Consanguineous Family

**DOI:** 10.3390/ijms22041549

**Published:** 2021-02-04

**Authors:** Laura Castilla-Vallmanya, Semra Gürsoy, Özlem Giray-Bozkaya, Aina Prat-Planas, Gemma Bullich, Leslie Matalonga, Mónica Centeno-Pla, Raquel Rabionet, Daniel Grinberg, Susanna Balcells, Roser Urreizti

**Affiliations:** 1 IBUB, IRSJD, and CIBERER (ISCIII), Department of Genetics, Microbiology and Statistics, Faculty of Biology, University of Barcelona, 08028 Barcelona, Spain; lcastilla30@gmail.com (L.C.-V.); aina.prat98@gmail.com (A.P.-P.); monicacentpla@hotmail.com (M.C.-P.); kelly.rabionet@ub.edu (R.R.); dgrinberg@ub.edu (D.G.); sbalcells@ub.edu (S.B.); 2 Department of Pediatric Genetics, Dr. Behcet Uz Children’s Hospital, Izmir 35210, Turkey; dr.semra@hotmail.com; 3 Department of Pediatric Genetics, Faculty of Medicine, Dokuz Eylul University, Izmir 35340, Turkey; ozlemgirayy@gmail.com; 4 CNAG-CRG, Centre for Genomic Regulation (CRG), Barcelona Institute of Science and Technology (BIST), 08028 Barcelona, Spain; gemma.bullich@cnag.crg.eu (G.B.); leslie.matalonga@cnag.crg.eu (L.M.)

**Keywords:** neurodevelopmental disease, clinical genetics, whole exome sequencing, consanguinity, focal dermal hypoplasia, holoprosencephaly

## Abstract

We present a Turkish family with two cousins (OC15 and OC15b) affected with syndromic developmental delay, microcephaly, and trigonocephaly but with some phenotypic traits distinct between them. OC15 showed asymmetrical skeletal defects and syndactyly, while OC15b presented with a more severe microcephaly and semilobal holoprosencephaly. All four progenitors were related and OC15 parents were consanguineous. Whole Exome Sequencing (WES) analysis was performed on patient OC15 as a singleton and on the OC15b trio. Selected variants were validated by Sanger sequencing. We did not identify any shared variant that could be associated with the disease. Instead, each patient presented a de novo heterozygous variant in a different gene. OC15 carried a nonsense mutation (p.Arg95*) in *PORCN*, which is a gene responsible for Goltz-Gorlin syndrome, while OC15b carried an indel mutation in *ZIC2* leading to the substitution of three residues by a proline (p.His404_Ser406delinsPro). Autosomal dominant mutations in *ZIC2* have been associated with holoprosencephaly 5. Both variants are absent in the general population and are predicted to be pathogenic. These two de novo heterozygous variants identified in the two patients seem to explain the major phenotypic alterations of each particular case, instead of a homozygous variant that would be expected by the underlying consanguinity.

## 1. Introduction

**Figure 1:**
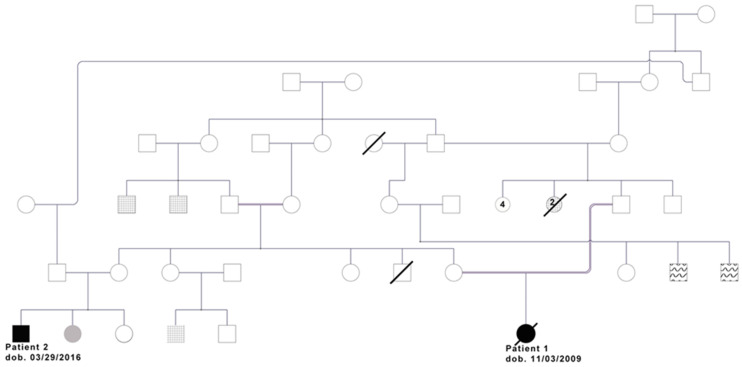
Extended family pedigree showing the relations among all four progenitors, the two mothers being sisters, and the two fathers being cousins once removed. Patient 1’s parents are also cousins once removed (connected through a double bond). Patients 1 and 2 in black. Patient 2’s microcephalic sister in grey. Squared pattern: congenital deafness. Wavy pattern: intellectual disability.

Case Description Here, we report two de novo mutations causing severe neurodevelopmental delay in two first degree cousins from a highly consanguineous family of Turkish origin (Figure 1).

**Figure 2:**
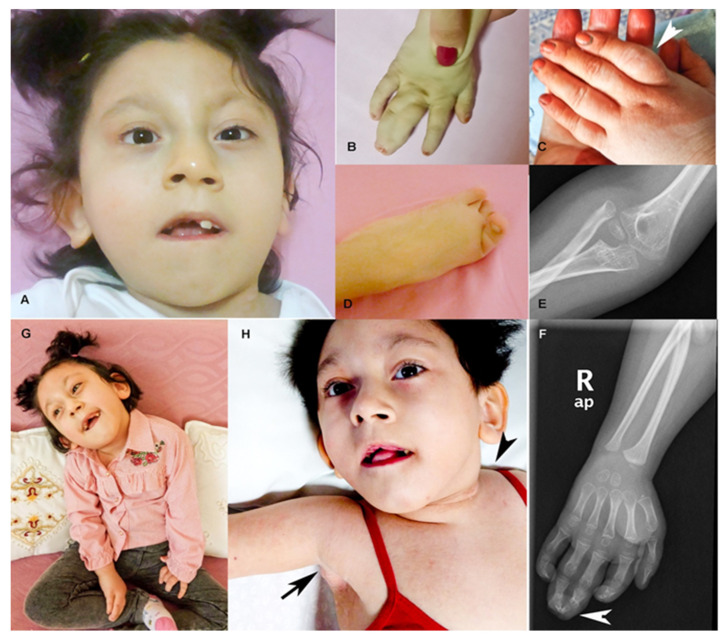
Pictures of patient 1. (**A**–**D**) Face, hand, and foot at age 9. Notice the asymmetry of the face, complete cutaneous syndactyly of the right second and third fingers, and overlapping toes. Thickened subcutaneous soft tissue on the proximal phalanges (prominently second finger) of the left hand (discoloration on the left hand due to traditional ‘red henna’). (**E**) Radial head dislocation. (**F**) Right hand radiograph showing distal phalangeal bone fusion of the third and fourth fingers at the age of 6. (**G**) Patient 1 at 9 years of age. Notice the generalized hypotonia. (**H**) Patient 1 at 6 years of age. Hyperemic, crusted skin lesions and focal dermal hypoplasia in her neck and axillary region and hypodontia are noticeable.

Patient 1 was a 10-year-old girl and single child of a healthy, consanguineous couple of Turkish origin. The family had a history of non-syndromic intellectual disability and deafness, with several affected individuals for each trait. Prenatally, the mother was hospitalized for a urinary tract infection. There was no history of polyhydramnios/oligohydramnios or maternal diabetes. Patient 1 was born at term by normal vaginal delivery and the birth was uneventful. Her birth weight and height were 3.1 kg (−0.53 SD) and 48 cm (−0.71 SD), respectively. Since birth, she presented with failure to thrive and neonatal hypotonia. Additionally, congenital hip dislocation was detected. At the six months follow-up, her weight was 4.5 kg (−4.08 SD), height was 58.5 cm (−3.07 SD), and head circumference was 37.3 cm (−4 SD). She showed several craniofacial dysmorphologies, including severe microcephaly, trigonocephaly (with prominent metopic ridge), facial asymmetry, and other features, such as low hairline, hypoplastic orbital ridges, up-slanting palpebral fissures, strabismus, sparse eyebrows, prominent ears, gingival hyperplasia, and high palate (Figure 2 and Table 1). Ocular examination revealed a minimal bilateral optic disc hypoplasia. Short neck, sometimes with crusting of the skin, was also observed. In addition, she presented with radial head dislocation, hypoplasia of the right clavicle, widely spaced nipples, ulnar deviation of the fingers, and complete cutaneous syndactyly of the right 3rd and 4th fingers with bony fusion of the distal phalanges, overlapping toes on both feet, and spasticity of the lower limbs. She had thickened subcutaneous soft tissue on the proximal phalanges (prominently second finger) of the left hand. The patient had focal dermal hypoplasia in the left region of the neck, axillary region, and the middle region of her right, lower limb. She had also hypo-hyperpigmented lesions in her back, pelvic region, and trunk (not shown). The patient also had hypodontia. Brain magnetic resonance imaging (MRI) revealed periventricular gliotic changes, thin corpus callosum, and moderate cerebellar atrophy. She was severely delayed (no head control at four years) and suffered from epilepsy. The seizures, which were generally triggered by fever, were amenable to multiple drug regimens. She passed away at the age of 10 due to a respiratory infection. She was considered compatible with Opitz C clinical spectrum. Previous genetic analyses included a normal karyotype and a normal array comparative genomic hybridization (array CGH).

**Figure 3:**
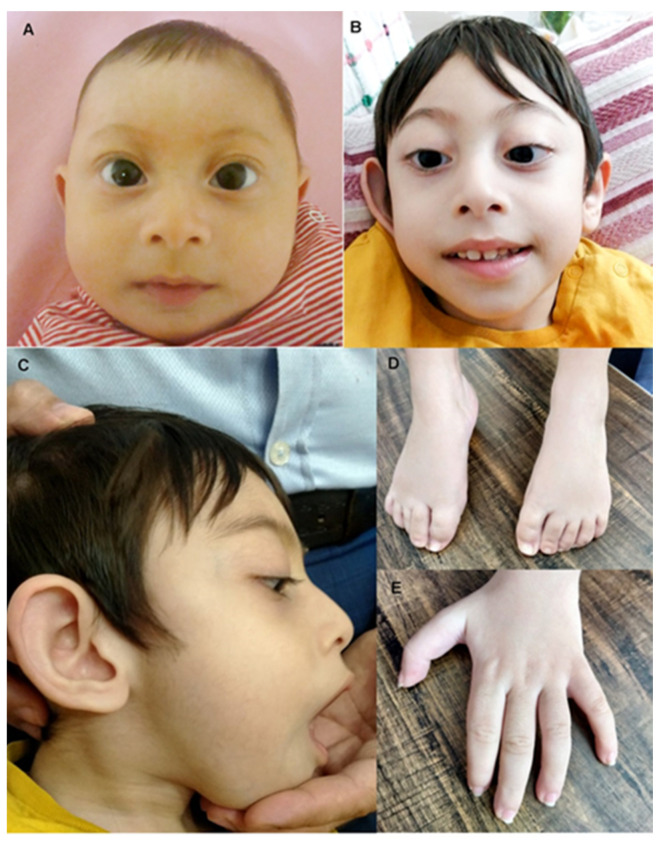
Distinctive features of patient 2. (**A**,**B**) Frontal view of the face at ages 8 months and 4 years. (**C**) Lateral view of the head at age 4. Microcephaly, retrognatia, ocular proptosis and upslanting palpebral fissures are appreciable. (**D**,**E**) Foot and hands of the patient at age 4.

Patient 2 was a first cousin of patient 1, sharing both the paternal and maternal lineages (Figure 3 and Table 1). He was a four-year-old boy and the third child of healthy, non-consanguineous Turkish parents. He was born at term by normal vaginal delivery with a weight of 3.08 kg (−0.72 SD). The mother had had one abortus because of ectopic pregnancy. One older sister (15 years old) presented with microcephaly (head circumference of 50 cm at 13 years, <1st percentile, −3.44 SD), without intellectual disability. The other sister (10 years old) was healthy. Patient’s 2 first examination was at 2 months. He was in the normal range for weight (4.8 kg, −0.88 SD) and height (58 cm, 0.04 SD), but he presented with microcephaly (Figure 3) with a head circumference under the first percentile (33 cm, −4.82 SD). At this time, his muscle tone was normal. Follow up at 8.5 months confirmed severe microcephaly (−4.58 SD), with semi-lobar holoprosencephaly and mild trigonocephaly shown by cranial MRI. Additional dysmorphologies included retrognathia, ocular proptosis, up-slanting palpebral fissures, prominent ears, mild tapering fingers, proximal placement of thumb, and short stature (but within the range of his close family). Eye, cardiac, and kidney examination at 8 months were normal. He presented with severe delay in developmental milestones, such as poor head control. Previous genetic analyses included normal karyotype and sequencing of KIF11, which showed no mutations. In spite of some clear differences with his cousin (Table 1), shared phenotypes suggested the presence of one common, major genetic cause for both patients.

## 2. Results and Discussion

Screening for shared pathogenic variants between the two cases was negative. Patient 1 showed large homozygous regions, as revealed by PLINK [1] analysis (55 fragments of more than 1 Mb of homozygosity, with a majority of fragments 2–6 Mb long), indicating consanguinity, while patient 2 showed 20 fragments of more than 1Mb of homozygosity (above average). Assuming an autosomal recessive inheritance pattern, patient 1 displayed variants in 52 selected genes, while patient 2 presented variants in 12 selected genes (Supplementary Tables S1 and S2). None of these homozygous variants was clearly pathogenic. In contrast, each patient harboured a de novo variant in a known ID gene. Patient 1 was heterozygous for a de novo, previously described [2], pathogenic variant in *PORCN* (2)(MIM * 300651), p.Arg95* (X:48369829 C>T; ENST00000326194: c.283C>T), while patient 2 carried a novel de novo heterozygous mutation at the Zinc-finger protein of the cerebellum 2, *ZIC2,* gene (MIM * 603073, mutation p.His404_Ser406delinsPro). Both mutations and their de novo status were confirmed by Sanger sequencing in the patients and their parents (and the respective cousin). WES and the Sanger sequencing results suggest that patient 1 is a mosaic for the *PORCN* p.Arg95* mutation.

The nonsense mutation p.Arg95* in *PORCN* creates a premature STOP codon at position 95 of the 461 residue-long protein-serine O-palmitoleoyltransferase porcupine protein. The mutation lies in exon 2 of 14, which is, therefore, subject to the degradation of the mutated mRNA by nonsense-mediated decay (NMD). In addition, if translated, the truncated protein would lack the majority of functional domains, including the active site. *PORCN* mutations have been associated with Goltz-Gorlin Syndrome (or focal dermal hypoplasia, FDH, MIM #305600), transmitted as an X-linked dominant trait, and the p.Arg95* mutation found in patient 1 had been identified in a 30 year-old woman also presenting with linear skin lesions, asymmetrical skeletal defects, clinodactyly, dental defects, and microcephaly [2]. However, there is no mention of developmental delay/intellectual disability (DD/ID). Goltz-Gorlin Syndrome is a clinical entity with high phenotypic heterogeneity and developmental delay, although rare, that is described in about 10%–15% of the patients [3,4,5]. In addition, patient 1 presented with the more frequently reported clinical characteristics, including facial asymmetry, short stature, agenesis of the corpus callosum, or moderate cerebellar atrophy. All this points to p.Arg95* in *PORCN* as a pathogenic mutation and the main cause of the disorder observed in patient 1. PORCN is a key regulator of the Wnt signalling pathway mediating the attachment of palmitoleate, a 16-carbon monounsaturated fatty acid, to Wnt proteins. Serine palmitoleylation of Wnt proteins is required for efficient binding to frizzled receptors and activation of Wnt signalling [6].

On the other hand, while patient 1 fits well in the FDH phenotypic entity, she has a more severe level of DD than other reported cases. Therefore, we hypothesized that other genetic factors might be contributing to her severe neurological presentation, especially since other family members present with non-syndromic ID (Figure 1). Worthy of interest, three variants were found in homozygosity in relevant genes (Supplementary Table S1): two missense variants in *NCAPD3* [c.3488C>T, p.(Pro1163Leu), rs143158496*] and in *YY1AP1* [c.1858G>A; p.(Ala620Thr)], and a synonymous variant with a putative effect on splicing in *UFC1* (c.246C>T, p.Ile82=). The variant in *NCAPD3*, is present in public databases at a very low frequency, although slightly higher in the Turkish population (gnomAD April 2020: 29 carriers out of 251446 alleles, no homozygotes, higher minor allele frequency (MAF) 0.0009 for East Asian, GME Variome: 1/163, MAF:0.006, Turkish population), which would be consistent with a recessive pattern of inheritance. *NCAPD3* has been previously related to microcephaly with moderate developmental delay [7], and a mouse model for *Ncapd2 (NCAPD3* partner) shows microcephaly as well. However, only 8 out of 18 in silico predictors consider this change as putatively damaging and, while the *NCAPD3* gene is constrained for loss of function (LoF) variants, it does not show constraint for missense mutations (o/e = 1.07 at gnomAD). Therefore, this change is classified as a variant of unknown significance (VUS), according to the American College of Medical Genetics (ACMG) guidelines [8], but we cannot rule out its potential contribution to the severity of the microcephaly and the DD traits of patient 1. Recessive LoF mutations in *YY1AP1* associate with Grange syndrome (GRNG, MIM #602531), whose presentation includes syndactyly and learning difficulties [9,10,11]. The variant is also present in public databases (gnomAD 18 carriers out of 141388, highest MAF 0.00013, absent in GME Variome). Finally, *UFC1* is associated with “neurodevelopmental disorder with spasticity and poor growth” (MIM #618076) [12] and the variant identified in patient 1 is present in public databases at a very low frequency (gnomAD: 2 alleles out of 251490).

ZIC2 is a transcription factor involved both in activation and repression, which plays a key role in the early stages of the central nervous system (CNS) development [13]. The *ZIC2* mutation detected in patient 2 involves the deletion of 7 bp and the insertion of 1 bp at the cDNA position 1211 [c.1211_1217delACCCCAGinsC (ENST00000376335)], leading to the substitution of the three residues at positions 404 to 406 by a proline (p.His404_Ser406delinsPro). This variant is not found in the general population (gnomAD, Iranome, and GME Variome) and residues 404 to 406 are fully conserved through the 28 species analysed (Supplementary Figure S1). This variant is classified as pathogenic following the ACMG guidelines [8]. The ZIC proteins are defined by the presence of a zinc finger domain that consists of five Cys2His2-type zinc fingers and this change is in a mutational hot-spot affecting the fifth C2H2-type Zinc Finger domain of the ZIC2 protein.

Autosomal Dominant (AD) mutations in *ZIC2* have been associated with holoprosencephaly 5 (HPE5, MIM #609637), characterized by severe neurological impairment, seizures, and characteristic dysmorphic facies. These traits are consistent with the patient’s clinical phenotype, who presented with semi-lobar holoprosencephaly and severe developmental delay (seizures have not been identified so far). While this specific mutation has not been previously reported, other non-frameshift mutations affecting the same domain have been identified in HPE5 patients [14,15,16,17], including the substitution of His 404 by Arg [15]. In conclusion, we have classified the *ZIC2* heterozygous mutation as likely pathogenic, and as the disease-causing mutation responsible for the major phenotypic alterations of the patient.

Other changes that can contribute to the patient’s phenotype have also been evaluated (Supplementary Table S2). In particular, the patient and his two sisters are homozygous for mutation p.Tyr1073Cys in *CFTR* (7:117251713 A>G, ENST00000003084: c.3218A>G, p.Tyr1073Cys). This gene encodes an epithelial ion channel that mediates the transport of chloride ions. Autosomal Recessive (AR) mutations in it are associated with cystic fibrosis (CF, MIM #219700), sweat chloride elevation without CF, and congenital bilateral absence of vas deferens (CBAVD, MIM #277180). The same mutation identified in this family had been previously identified in a patient with neonatal hypertrypsinaemia without lung disease and is included in the Cystic Fibrosis Mutation database. This mutation is absent in the common population (gnomAD, Iranome, and GME Variome) and it is predicted to be damaging for the protein performance by most functional prediction algorithms. We have considered this variant to be likely pathogenic, according to the ACMG guidelines [8], and have recommended detailed clinical assessment of the patient and his sisters. In addition to the de novo *ZIC2* mutation, Patient 2 is a homozygote for the *KPNA7* p.Arg217Trp mutation (7:98786174 G>A, ENST00000327442: c.649C>T, Supplementary Table S2), inherited from his parents (both heterozygotes). The healthy sister does not carry the mutant allele, while the sister affected by microcephaly is also homozygous for this change. This variant is rare in the common population (only 27 carriers, no homozygotes, in gnomAD) and algorithms developed to predict the effect of missense changes on protein structure and function (SIFT, PolyPhen2, PROVEAN, or Mutation assessor) do not agree on the potential impact of this missense change. *KPNA7* recessive mutations have been associated with infantile spasms and cerebellar malformation only once in the literature [18]. These authors presented a pair of sibs affected with severe developmental disability, infantile spasms, intractable epilepsy consistent with Lennox–Gastaut syndrome, partial agenesis of the corpus callosum, and cerebellar vermis hypoplasia in which two heterozygous *KPNA7* mutations have been identified by WES. In these patients, microcephaly was not present (with head circumference at the 98th and 50th percentiles). In conclusion, this variant is classified as likely benign and does not seem to be related to the patient’s clinical phenotype, but its implication on moderate microcephaly cannot be ruled out without further studies.

Both causal genes that mutated in these cousins (*PORCN* and *ZIC2*) encode proteins that are directly implicated in the Wnt pathway. The mechanistic coincidence on these two factors could explain some of the clinical similarities among these patients, both harbouring mutations in genes that, prima facie, seem not to be related. Wnt signalling pathways regulate complex normal biological processes such as cell differentiation, development, tissue homeostasis, and wound healing. However, when the Wnt pathway is aberrantly regulated, it can be associated with bone anomalies, neurodevelopmental disorders, cancer, and cardiovascular diseases, among other diseases [19]. Wnt signalling is a particularly complex pathway that leads to the activation of two main, alternative branches: the canonical and the noncanonical pathway. In the canonical pathway, the binding of Wnt proteins to their receptors (Frizzled and Lrp5/6) triggers the inactivation or disassembly of the destruction complex, which reduces β-catenin phosphorylation and promotes its accumulation and translocation to the nucleus. There, β-catenin forms a complex with Lef/Tcf factors and induces the transcription of specific genes. Activation of the noncanonical pathway does not depend on β-catenin-driven transcription. Instead, it relies on changes that affect cytoskeletal organization and calcium homeostasis [19]. PORCN (protein-serine O-palmitoleoyltransferase porcupine) mediates palmitoylation of Wnt proteins, which is an essential modification necessary for their correct secretion and binding to the Frizzled receptors, thus, being a major regulator of Wnt signalling [20,21,22]. On the other hand, *Zic* genes are implicated in development of the dorsal neural tube and neural crest as well as the somites and the cerebellum. In addition to the regulation of multiple neural factors, ZIC2 functions as a modulator of Wnt signalling by the direct interaction with TCF4 [23] or by modulating β-catenin accumulation and activity [24].

## 3. Materials and Methods

Patient 1 was included in the URDCat genetic program and underwent whole exome sequencing (WES) analysis as previously described [25,26] (briefly, sequencing was done at the CNAG facility using the Nimblegen SeqCap EZ MedExome + mtDNA 47Mb capture kit aiming at 90× coverage). Patient 2 was analyzed by trio-WES, briefly, (samples from patient 2 and his parents were also sequenced at the CNAG facility). In this case, capture was performed with Agilent SureSelect v5 (Agilent, CA, USA). The samples were sequenced at a coverage of 140×. Basic bioinformatic processing of the sequencing data was performed using CNAG’s in-house pipeline. Annotation and filtering for both cases was performed with VarAFT [26]. All variants included in Tables S1 and S2 were analysed by Sanger sequencing in the patient, mother, and father, as well as in her affected cousin. Primers and conditions used for PCR amplification and sequencing are available on demand. Sequence alterations are reported according to the Human Genome Variation Society (HGVS) nomenclature guidelines. The gnomAD (v.2.1.1), Iraniome, and Great Middle East Variome databases were accessed on May 2020.

## 4. Conclusions

Two main considerations arise from this study. In the first place, while in Western countries syndromic and non-syndromic DD and ID are mainly sporadic and caused by de novo mutations [27], in the near and Great Middle East, where there is a high consanguinity rate [28], ID is commonly due to recessive mutations [29]. When presented with highly consanguineous families segregating a disease phenotype, one of the first approaches is to search for pathogenic mutations in homozygosity tracks. In addition, performing WES analysis only on the index case is a common practice, which, in highly consanguineous populations, yields acceptable diagnostic results [30]. While the cost advantage of such an approach is evident, it is important to keep in mind that, even in cases with a very high consanguinity, de novo disease causing mutations may still be present, representing up to 27.8% of the ID mutations identified in these families [29] when trio-WES is performed. It should not be forgotten that these mutations are still one of the major causes of ID. Thus, to avoid misdiagnosis, we strongly recommend trio-WES or, if unavailable, a cautious revision of putative dominant mutations (likely followed by segregation analysis of a higher number of putative candidates) even in these families. In the present study, WES of patient 1 was followed by segregation analysis of up to 16 heterozygous variants, while the trio-WES of patient 2 involved further checking of only six variants. Otherwise, we risk basing our diagnosis solely on putatively pathogenic homozygous variants in the detriment of undetected de novo mutations also present in the patient.

The second important consideration from this study is the co-occurrence of multiple genetic diseases in the same individual, leading to a very complex phenotype. This can be especially likely in families with high levels of consanguinity, potentially segregating various pathogenic mutations [31].

## Figures and Tables

**Table 1:** Summary of the clinical features observed in each patient.

Phenotype	HPO Term	Patient 1	Patient 2
Microcephaly	HP:0000252	+	+
Trigonocephaly	HP:0000243	+	+
Protruding ears	HP:0000411	+	+
Upslanted palpebral fissure	HP:0000582	+	+
Neurodevelopmental delay	HP:0012758	+	+
Short stature	HP:0004322	+	+
Muscular hypotonia	HP:0001252	+	+
Low anterior hairline	HP:0000294	+	−
Facial asymmetry	HP:0000324	+	−
Sparse eyebrows	HP:0045075	+	−
Underdeveloped supraorbital ridges	HP:0009891	+	−
Strabismus	HP:0000486	+	−
High palate	HP:0000218	+	−
Hypodontia	HP:0000668	+	−
Gingival overgrowth	HP:0000212	+	−
Failure to thrive	HP:0001508	+	-
Dislocated radial head	HP:0003083	+	−
Ulnar deviation of finger	HP:0009465	+	−
3-4 finger syndactyly	HP:0006097	+	−
Hip dislocation	HP:0002827	+	−
Hypopigmented skin patches	HP:0001053	+	−
Focal dermal hypoplasia	HP:0007510	+	−
Seizures	HP:0001250	+	−
Semilobar holoprosencephaly	HP:0002507	−	+
Proptosis	HP:0000520	−	+
Retrognathia	HP:0000278	−	+
Tapered fingers (mild)	HP:0001182	−	+

## Data Availability

The data that support the findings of this study are available on request from the corresponding author. The data are not publicly available due to privacy and ethical restrictions.

## Supplementary Materials

The following are available online at https://www.mdpi.com/1422-0067/22/4/1549/s1.
