# Supplementary material for: De Novo PORCN and ZIC2 Mutations in a Highly Consanguineous Family
Source: Int J Mol Sci. 2021 Feb 4;22(4):1549. doi: 10.3390/ijms22041549 (PMC7913830; doi:10.3390/ijms22041549)
Supplement: Supplementary file 1 [file ijms-22-01549-s001.zip › Suppl Files/Supl. Figure 1.pdf]

DE NOVO PORCN AND *ZIC2* MUTATIONS IN A HIGHLY CONSANGUINEOUS FAMILY

Castilla-Vallmanya, Laura[1]; Gürsoy, Semra[2]; Giray-Bozkaya, Özlem[3]; Prat-Planas, Aina[1]; Bullich, Gemma[4]; Matalonga, Leslie[4]; Centeno-Pla, Monica[1]; Rabionet, Raquel[1]; Grinberg, Daniel[1]; Balcells, Susanna[1]; Urreizti, Roser[1*].

```
                                          404-406
ZIC2_HUMAN O95409-1          PYLCKM--CDKSYTHPSSLRKHMKVHESSPQGSESSPAASSGYESSTPPGLVSPSAEPQS  449
[Pongo_abelii]               PYLCKM--CDKSYTHPSSLRKHMKVHESSPQGSESSPAASSGYESSTPPGLVSPSAEPQS  449
[Macaca_mulatta]             PYLCKM--CDKSYTHPSSLRKHMKVHESSPQGSESSPAASSGYESSTPPGLVSPSAEPQS  449
[Cebus_imitator]             PYLCKM--CDKSYTHPSSLRKHMKVHESSPQGSESSPAASSGYESSTPPGLVSPSSEPQS  449
[Canis_lupus_dingo]          PYLCKM--CDKSYTHPSSLRKHMKVHESSPQGSESSPAASSGYESSTPPGLVSPSAEPQS  448
[Ursus_arctos_horribilis]    PYLCKM--CDKSYTHPSSLRKHMKVHESSPQGSESSPAASSGYESSTPPGLVSPSAEPQS  448
[Rattus_norvegicus]          PYLCKM--CDKSYTHPSSLRKHMKVHESSPQGSESSPAASSGYESSTPPGLVSPSAEPQS  448
[Mus_musculus]               PYLCKM--CDKSYTHPSSLRKHMKVHESSPQGSESSPAASSGYESSTPPGLVSPSAEPQS  448
[Felis_catus]                PYLCKM--CDKSYTHPSSLRKHMKVHESSPQGSESSPAASSGYESSTPPGLVSPSAEPQS  448
[Camelus_ferus]              PYLCKM--CDKSYTHPSSLRKHMKVHESSPQGSESSPAASSGYESSTPPGLVSPSAEPQS  448
[Sus_scrofa]                 PYLCKM--CDKSYTHPSSLRKHMKVHESSPQGSESSPAASSGYESSTPPGLVSPSAEPQS  448
[Ovis_aries]                 PYLCKM--CDKSYTHPSSLRKHMKVHESSPQGSESSPAASSGYESSTPPGLVSPSAEPQS  448
[Vulpes_vulpes]              PYLCKM--CDKSYTHPSSLRKHMKSPLVSPPGFCLAGP----------------------  426
[Bos_taurus]                 PYLCKM--CDKSYTHPSSLRKHMKVHESSPQGSESSPAASSGYESSTPPGLVSPSAEPQS  448
[Parus_major]                PYLCKM--CDKSYTHPSSLRKHMKVHESSPQGSESSPAASSGYESSTPPGLVSPSAESQS  425
[Falco_rusticolus]           PYLCKM--CDKSYTHPSSLRKHMKVHESSPQGSESSPAASSGYESSTPPGLVSPSAESQS  425
[Chelonia_mydas]             PYLCKM--CDKSYTHPSSLRKHMKVHESSPQGSESSPAASSGYESSTPPGLVSPSAETQS  424
[Gallus_gallus]              PYLCKM--CDKSYTHPSSLRKHMKVHESSPQGSESSPAASSGYESSTPPGLVSPSAEPQS  423
[Suricata_suricatta]         PYLCKM--CDKSYTHPSSLRKHMKSPLVSPPGFCLAGP----------------------  370
[Labeo_rohita]               PYLCKM--CDKSYTHPSSLRKHMKVHESSPSASDSSPAASSGYESSTPPGLVSPSTETQS  417
[Denticeps_clupeoides]       PYLCKM--CDKSYTHPSSLRKHMKVHESSPSASDSSPAASSGYESSTPPGLVSPSTETQS  417
[Danio_rerio]                PYLCKM--CDKSYTHPSSLRKHMKVHESSPPASDSSPAASSGYESSTPPGLVSPSTETQS  417
[Takifugu_rubripes]          PYLCKM--CDKSYTHPSSLRKHMKVHESSPSASDSSPAASSGYESSTPPGLVSPTTETQS  474
[Podarcis_muralis]           PYLCKM--CDKSYTHPSSLRKHMKVHESSPQGSESSPAASSGYESSTPPGLVSPSAETQS  455
[Xenopus_tropicalis]         PYLCKM--CDKSYTHPSSLRKHMKVHESSPQGSESSPAASSGYESSTPPGLVSPNSETQN  468
[Xenopus_laevis]             PYLCKM--CDKSYTHPSSLRKHMKVHESSPQGSESSPAASSGYESSTPPGLVSPNSETQN  468
[Caenorhabditis_elegans]     PYSCMYPDCGKTYTHPSSLRKHTKVHENEKKSQLSPEHDESS-DSGNA-SIGTPTTD--E  252
[Drosophila_melanogaster]    PYNCRINGCDKSYTHPSSLRKHMKVHGNVDEKSPSHGYDSEGEESSSSSII-TGGAQTPP  408
                             ** *    *.*:********** *
```

Supplementary Figure 1. Alignment of the region of interest (from the human residue 392 to 449) of ZIC2 proteins in multiple vertebrates, *C. elegans* and *D. melanogaster*. Highlighted in yellow residues 404 to 406, belonging to the 5th C2H2-type Zinc Finger, substituted by proline in Patient 2.
